# Circulating exosomal gastric cancer-associated long noncoding RNA1 as a noninvasive biomarker for predicting chemotherapy response and prognosis of advanced gastric cancer: A multi-cohort, multi-phase study

**DOI:** 10.1016/j.ebiom.2022.103971

**Published:** 2022-03-27

**Authors:** Qiying Song, Xiaohui Lv, Yi Ru, Jian Dong, Rongyan Chang, Di Wu, Lubin Chen, Xinxin Wang, Xin Guo

**Affiliations:** a Department of Digestive Surgery, Xijing Hospital, Fourth Military Medical University, Xi'an, 710032, China; b Department of Endoscopic Surgery, Air Force 986^th^ Hospital, Fourth Military Medical University, Xi'an, 710032, China; c Department of General Surgery, Chinese PLA General Hospital, Beijing, 100853, China.; d Department of Gynecology and Obstetrics, Xijing Hospital, Fourth Military Medical University, Xi'an, 710032, China; e Department of Biochemistry and Molecular Biology, Fourth Military Medical University, Xi'an, 710032, China

**Keywords:** Exosome, LncRNA-GC1, Adjuvant chemotherapy, Prognosis, Gastric cancer

## Abstract

**Background:**

A previous validated study has identified the diagnostic value of circulating exosomal lncRNA-GC1 for detecting and monitoring gastric cancer. We aimed to further determine the predictive role of circulating exosomal lncRNA-GC1 for prognosis and chemotherapy response.

**Methods:**

We retrospectively conducted a multi-phase analysis with four independent cohorts of 981 patients. A training cohort was used to generate the predictive model. One internal and two external cohorts were recruited as validation cohorts. Patients with stage II or III gastric cancer in the combined cohort were used to evaluate the predictive value of circulating exosomal lncRNA-GC1 for chemotherapy response.

**Findings:**

In the training cohort, circulating exosomal lncRNA-GC1 was identified as an independent prognostic predictor for disease-free and overall survival. A prognostic risk stratification model based on circulating exosomal lncRNA-GC1 and AJCC stage revealed better predictive accuracy for disease-free and overall survival than the traditional AJCC stage system alone (C-index: DFS 0.701 vs 0.614; OS 0.720 vs 0.611, both P<0.05). And it has been further verified in the validation cohorts. In interaction analysis, for stage II and III GC, patients with low-level of circulating exosomal lncRNA-GC1 derived more survival benefit from adjuvant chemotherapy (P < 0.05); while those with high-level did not.

**Interpretation:**

Measurement of circulating exosomal lncRNA-GC1 provides clinically important prognostic information and could complement the AJCC stage to optimize decision-making for selecting patients who could benefit more from fluorouracil-based chemotherapy after surgery.

**Funding:**

The funders are listed in the Acknowledgement.

**Research in context**

**Evidence before this study**

Studies have shown that GC-associated long noncoding RNA1 (lncRNA-GC1) functions as a scaffold by binding histone acetyltransferases WDR5 and KAT2A, which then promote progression of GC. Our previous study has identified lncRNA-GC1 as an abnormally high-expressed and GC-specific lncRNA both in GC cells and exosomes, and validated the promising efficacy of circulating exosomal lncRNA-GC1 for early detection and monitoring progression of GC.

**Added value of this study**

Our results demonstrated that high-level circulating exosomal lncRNA-GC1 was an independent and unfavorable prognostic predictor for GC. A new prognostic risk stratification model based on circulating exosomal lncRNA-GC1 and AJCC stage revealed satisfied predictive accuracy. In addition, for advanced GC, patients with low-level of circulating exosomal lncRNA-GC1 may derived more survival benefit from adjuvant chemotherapy

**Implications of all the available evidence**

Measurement of circulating exosomal lncRNA-GC1 provides clinically important prognostic information and could complement AJCC stage to optimize decision making for selecting patients who could benefit more from fluorouracil-based chemotherapy after surgery.

Alt-text: Unlabelled box

## Introduction

The incidence and mortality of gastric cancer (GC) have seen a steady decline in most western countries.^1^^,^^2^ However, it still ranks the second leading cause of cancer-related deaths in Asian countries, especially in China, Japan and South Korea.^2^^,^^3^ As the current standard treatment strategy, adjuvant chemotherapy after surgery is generally advised for patients with advanced GC.^4^ Over the past 40 years, fluorouracil-based regimens remain the first-line choice for adjuvant chemotherapy. However, several classic clinical trials have indicated that the overall survival of patients in the adjuvant chemotherapy group was only a slightly increased than that in surgery-only group, suggesting that not all patients benefit from adjuvant chemotherapy.^5^^,^^6^ These findings strongly highlight the urgent need for further classification of GC, the identification of patients at different risk of recurrence and the determination of likelihood for chemotherapy benefit.

With the widespread applications of high-throughput sequencing, large sets of genomic information of various cancers have been gained. The biological complexity of cancer phenotypes has also been systematically analyzed. Disease-specific biomarkers have been developed to identify particular cancer phenotypes. For colorectal cancer and breast cancer, some specific molecular subtypes have already been shown with promising predictive value for individualized prognosis and chemotherapy benefit.^7^, ^8^, ^9^, ^10^, ^11^, ^12^ For gastric cancer, although several potential biomarkers have been identified to have predictive value for prognosis, because of the absence of external validation cohorts, molecular characteristics for predicting individualized prognosis and chemotherapy benefit have not been fully validated.^13^, ^14^, ^15^, ^16^, ^17^, ^18^, ^19^, ^20^

Exosomes, secreted by viable cells, have been verified to show specific information from their cells of origin.^21^^,^^22^ Studies have shown that GC-associated long noncoding RNA1 (lncRNA-GC1) functions as a scaffold by binding histone acetyltransferases WDR5 and KAT2A, which then promote progression of GC.^23^ Based on high-throughput sequencing, our previous study has identified lncRNA-GC1 as an abnormally high-expressed and GC-specific lncRNA both in GC cells and exosomes.^24^ More importantly, we have also validated the promising efficacy of circulating exosomal lncRNA-GC1 for early detection and monitoring progression of GC, and the stability of lncRNA-GC1 packaged in exosomes meets the needs of laboratory testing.^24^ However, the role of circulating exosomal lncRNA-GC1 in predicting chemotherapy response and prognosis is poorly understood.

Here we performed this multi-cohort, multi-phase study to determine whether circulating exosomal lncRNA-GC1 could serve as an independent prognostic factor for patients with resectable GC. In addition, we systematically investigated the predictive value of a prognostic risk stratification model based on circulating exosomal lncRNA-GC1 with three independent validation cohorts. Finally, we identified circulating exosomal lncRNA-GC1 as an indicator of fluorouracil-based chemotherapy benefit. Our ultimate goal was to determine a noninvasive biomarker for predicting treating response and select patients who may benefit from chemotherapy.

## Methods

### Patients and specimens

In the discovery phase, a training cohort of 375 GC patients was consecutively recruited from the Air Force 986th Hospital between December 2012 and December 2015 for model establishment. In the validation phase, three independent cohorts were recruited with one internal validation cohort from the Air Force 986th Hospital (n=262) between December 2014 and February 2016 and two additional external validation cohorts from Xijing Hospital (n=186) and Chinese PLA General Hospital (n=158) December 2014 and May 2016. Participants in two phases were enrolled according to the enrollment criteria. The inclusion criteria were as follows: confirmation of histological gastric adenocarcinoma; treatment with standard radical gastrectomy (>15 lymph nodes harvested); administration of fluorouracil-based adjuvant chemotherapy for at least 4 cycles or no adjuvant chemotherapy; available of detailed clinicopathological characteristics and follow-up data. The exclusion criteria were as follows: preoperative anticancer treatments including chemotherapy, radiotherapy, immunotherapy or other cytotoxic therapy; postoperative anticancer treatments in addition to routine chemotherapy; tumors other than gastric cancer; postoperative death due to complications within one month. Serum samples were obtained one week before surgery.

The detailed clinicopathological characteristics of each participant, including gender, age, tumor location, differentiation status, Lauren type and the AJCC stage system were retrospectively collected. Differentiation status was classified as well, moderate, poor and undifferentiated according to the World Health Organization's gastric cancer treatment guidelines.^25^ The clinical staging was determined according to the 8th edition of the American Joint Committee on Cancer (AJCC) and the International Union Against Cancer tumor-node-metastasis (TNM) staging system.^26^ Disease-free survival refers to the time from operation to the date of disease progression or death. Overall survival refers to the time from operation to the last follow-up or death. Data were analyzed between January 2021 and June 2021.

### Ethics

This study was approved by the Ethics Committees of the three participating organizations: Chinese PLA General Hospital (S2019-334-01), Air Force 986th Hospital (AF986-LL202003182-1), and Xijing Hospital (KY202001301-F-1). Informed consent from all participants were obtained before enrollment.

### Exosome's isolation and characterization

The isolation and characterization of circulating exosomes were carried out as previously reported.^27^ Briefly, exosomes in plasma samples were passed through a 0.20-μm membrane filter (Millipore) followed by concentration with ultracentrifugation. The isolated particles were identified with transmission electron microscopy (Thermo Scientific) and NanoSight (NS300, UK) for size and distribution. As for confirmation of exosomes’ surface markers by western blot, proteins were extracted with Radio Immunoprecipitation Assay (RIPA) buffer, concentrated with sodium dodecyl sulfate (SDS), transferred to a polyvinylidene fluoride (PVDF) membrane and incubated with the primary antibodies as follows: anti-CD9 (1:2000; CST, USA), anti-CD63 (1:1000; CST, USA), and anti-tubulin (1:2000; CST, USA) at 4°C overnight for at ≥8 h).

### Measurement of exosomal lncRNA-GC1 and cutoff value selection

The total plasma-derived exosomal RNA was extracted according to the Trizol method. cDNA was synthesized from 30 ng of isolated RNA with a MMLV kit (Takara, Japan). Polymerase Chain Reaction was amplificated with denaturation at 95°C for 5 min, followed by 40 cycles of at 95°C for 10 s, and 60°C for 30 s. The relative levels of circulating exosomal lncRNA-GC1 were normalized using the 2^−ΔΔCt^ method. The sequences of primers used for amplifying lncRNA-GC1 were as follows: sense: F-TGGGGTAACTTAGCAGTTTCAAT-R; antisense: F-GGCAAGCAGTAATCTTACATGACAC-R. The optimal cut-off value of lncRNA-GC1 for prognosis was determined by maximizing the Youden index of the receiver operating characteristic curve (ROC). The stability of exosomal lncRNA-GC1 has been previously identified to remain constant as the total circulating lncRNA-GC1 was packaged within exosomes.^24^ The time from collection to measurement was within 12 hours after serum collection.

### Statistical analysis

Continuous data were presented as the mean (± standard deviation, SD) with ANOVA tests if normally distributed or as the median (interquartile range, IQR) with Kruskal-Wallis tests if not normally distributed. Categorical data were presented as proportions and percentages and were evaluated using the chi-square test or Fisher's exact test. Survival analysis of patients’ subgroups was compared by the Kaplan-Meier survival curve with the log-rank test. Univariable analyses were applied to estimate the hazard ratio (HR) with 95% confidence intervals (CI), and the factors with a P value less than 0.05 in the univariable analyses were included in the multivariable analysis to identify the independent prognostic factor by Cox proportional hazards models. The predictive model was constructed based on the known clinical prognostic factors and availability based on independent risk factors.^28^ After the test of the Cox proportional hazards assumption, this model was implemented into the nomogram. The model's performance of discrimination was evaluated by the Concordance index (C-index) and the area under the receiver operating characteristic curve (AUC) with Delong's test.^29^^,^^30^ Calibration plots were used to evaluate the goodness of fit between the predicted probabilities and observed outcomes. Interactions between the lncRNA-GC1 levels and postoperative adjuvant chemotherapy treatment were also detected by the multivariable Cox model. All statistical analyses were conducted with SPSS software (SPSS 26.0), RStudio software (RStudio,1.2.5033) with R packages of “rms”, “time ROC”, “pROC” and “compareC”. Differences with two-sided P<0.05 were considered statistically significant.

### Role of funding source

The funders had no role in the study design, data collection, data analyses, interpretation, paper writing or publishing decision. The corresponding authors had full access to all the data in this study and had full responsibility for the decision to submit for publication.

## Results

### Study design

**Figure 1:**
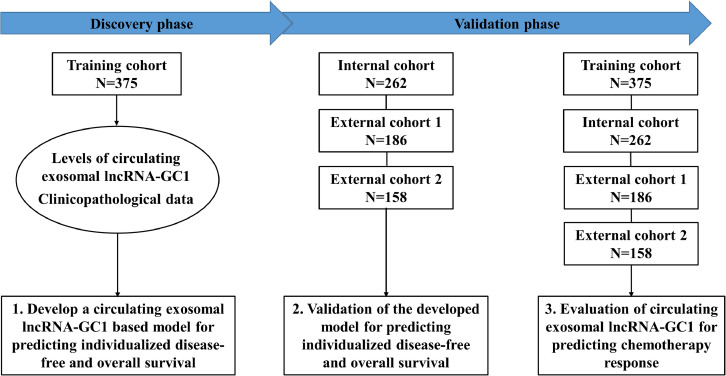
**The flowchart for the training and validation of a predictive model based on circulating exosomal lncRNA-GC1.** The levels of circulating exosomal lncRNA-GC1 were measured in four different cohorts during three phases. In discovery phase, the training cohort was used to develop a predictive model based on circulating exosomal lncRNA-GC1 and clinicopathological data. In validation phase, the model was validated in one internal and two external cohorts. In interaction phase, patients with stage II or III gastric cancer in combined cohort were used to assess the association between circulating exosomal lncRNA-GC1 and chemotherapy response.

Our previous study has shown promising efficacy of circulating exosomal lncRNA-GC1 for early detection and monitoring progression of gastric cancer.^24^ Based on previous findings, we hypothesize that circulating exosomal lncRNA-GC1 will also allow noninvasive prediction of individualized prognosis and chemotherapy benefits for patients with gastric cancer. To this end, we conducted this multi-cohort, multi-phase study. The flow diagram is shown in Figure 1. The majority of total patients (n=814, 83.0%) had stage II or III GC, among whom 476 (58.5%) patients received postoperative fluorouracil-based adjuvant chemotherapy. The detailed clinicopathological characteristics of patients in four cohorts were similar and given in Table 1.

**Table 1:** Characteristics of patients in training, internal and external validation cohorts.

Factors	Training cohort (n=375)		Internal validation cohort (n=262)		External validation cohort 1 (n=186)		External validation cohort 2 (n=158)		P value
	N	%	N	%	N	%	N	%	/
Age (Median, IQR)	56 (51-66)		58 (50-65)		59 (49-67)		59 (50-68)		0.245*
Gender Female Male	119 256	31.7 68.3	93 169	35.5 64.5	71 115	38.2 61.8	56 102	35.4 64.6	0.466
Tumor location Cardia Body Antrum Whole	75 61 189 50	20.0 16.3 50.4 13.3	58 33 130 41	22.1 12.6 49.6 15.6	43 30 93 20	23.1 11.6 50.0 10.8	26 23 85 24	16.5 14.6 53.8 15.2	0.697
Differentiation status Well + Moderate Poor + undifferentiated	128 247	34.1 65.9	74 188	28.2 71.8	52 134	28.0 72.0	49 109	31.0 69.0	0.326
Lauren type Intestinal Diffuse or mixed	271 104	72.3 27.7	201 61	76.7 23.3	137 49	73.7 26.3	110 48	69.6 30.4	0.412
Depth of invasion pT_1_ pT_2_ pT_3_ pT_4a_ pT_4b_	41 26 130 145 33	10.9 6.9 34.7 38.7 8.8	18 20 100 112 12	6.9 7.6 38.2 42.7 4.6	16 12 61 87 10	8.6 6.5 32.8 46.8 5.4	17 9 61 61 10	10.8 5.7 38.6 38.6 6.3	0.423
Lymph node metastasis pN_0_ pN_1_ pN_2_ pN_3a_ pN_3b_	133 52 91 45 54	35.5 13.9 24.3 12.0 14.4	81 40 65 31 45	30.9 15.3 24.8 11.8 17.2	58 27 47 22 32	31.2 14.5 25.3 11.8 17.2	48 33 37 23 17	30.4 20.9 23.4 14.6 10.8	0.656
Metastasis pM_0_ pM_1_	362 13	96.5 3.5	256 6	97.7 2.3	184 2	98.9 1.1	152 6	96.2 3.8	0.318
AJCC stage I II III IV	58 91 213 13	15.5 24.3 56.8 3.5	34 56 166 6	13.0 21.4 63.4 2.3	26 41 117 2	14.0 22.0 62.9 1.1	22 40 90 6	13.9 25.3 57.0 3.8	0.664
Chemotherapy No Yes	181 184	48.3 51.7	142 120	54.2 45.8	95 91	51.1 48.9	77 81	48.7 51.3	0.493

Note: * means Kruskal-Wallis test.

### Correlations between circulating exosomal lncRNA-GC1 and clinicopathological characteristics

In the discovery phase, the training cohort was used to determine the prognostic cut-off value of circulating exosomal lncRNA-GC1 to classify high versus low levels. As shown in eFigure 1 in the Supplement, the optimal cut-off value of lncRNA-GC1 was determined as 15.0 by the maximum Youden index according the respective receiver operating characteristic (ROC) curves for overall survival in the training cohort. Based on the optimum cut-off value of 15.0, patients were divided into two groups (high-level group vs low-level group). The high-level group included 231 patients (61.6%) and the low-level group included 144 patients (38.4%). The levels of circulating exosomal lncRNA-GC1 were significantly correlated with T, N and AJCC stage (P<0.05), other than other clinicopathological characteristics (eTable 1). The levels of circulating exosomal lncRNA-GC1 were also significantly correlated with T, N and AJCC stage (P<0.05), other than other clinicopathological characteristics in validation cohorts (eTable 1).

These results suggest that circulating exosomal lncRNA-GC1 showed strong correlation with tumor burden and had no correlations with clinicopathological characteristics other than TNM stage system that may influence the following predictive model.

### Correlations between circulating exosomal lncRNA-GC1 and prognosis

**Figure 2:**
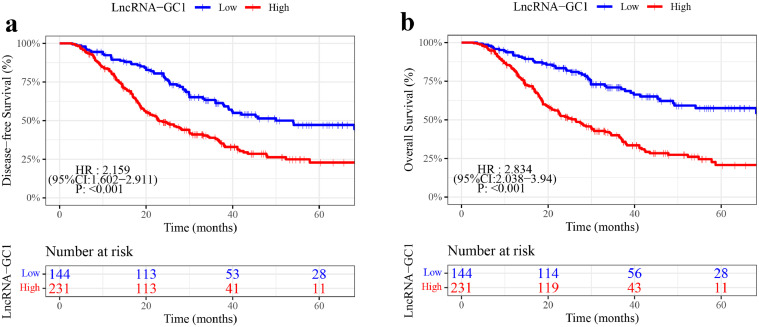
Kaplan-Meier analyses of DFS and OS according to the levels of circulating exosomal lncRNA-GC1 in patients with gastric cancer in the training cohort (n=375). (a) DFS, disease-free survival. (b) OS, overall survival. HR, hazard ratio. CI, confidence interval.

Then, we investigated the prognostic value of circulating exosomal lncRNA-GC1. In discovery phase, Kaplan-Meier analysis indicated that patients with low-level lncRNA-GC1 showed more disease-free and overall survival benefit than those with high-level (DFS, HR, 2.159, 95%CI, 1.602-2.911, P<0.001; OS, HR, 2.834, 95%CI, 2.038-3.94, P<0.001, Figure 2) in training cohort. Moreover, in validation phase, these findings were also confirmed in the internal, external validation and combined cohorts (eFigure 2 a-d).

As for stratified analysis of prognosis, in training cohort, patients with either early stage (stage I) or advanced stage (stage II and III), those with low-level lncRNA-GC1 revealed better disease-free and overall survival benefit than those with high-level (P<0.05) (eFigure 3 a and eFigure 4 a). These findings were further validated in internal cohort, external cohort 1 and external cohort 2 (eFigure 3 b-d and eFigure 4 b-d). Importantly, lncRNA-GC1 retained a powerful prognostic predictor after stratification by age, gender, tumor location, Lauren type, differentiation status, pT stage and pN stage in training, internal and external cohorts (eFigure 5-12). These results strongly suggest that circulating exosomal lncRNA-GC1 was significantly correlated with prognosis of patients with GC independent of clinical data.

### Circulating exosomal lncRNA-GC1 as an independent prognostic factor

To further investigate whether circulating exosomal lncRNA-GC1 was correlated with prognosis, we performed univariate analysis with training cohort. Age, AJCC stage, and circulating exosomal lncRNA-GC1 were significantly correlated with disease-free and overall survival (P<0.05) (eTable 2). Furthermore, as shown by multivariate analysis, AJCC stage and circulating exosomal lncRNA-GC1 were both identified as independent prognostic factors for gastric cancer (P<0.05) (eTable 2). Moreover, in validation phase, circulating exosomal lncRNA-GC1 remained its prognostic value in internal, external 1 and external 2 cohorts by Univariate and multivariate analyses (eTable 3-5). These results suggest that circulating exosomal lncRNA-GC1 serves as an independent prognosticator for gastric cancer.

### Prognostic value of circulating exosomal lncRNA-GC1 based predictive model

First, as for circulating exosomal lncRNA-GC1 and AJCC stage have been proved to be two independent prognostic factors, we tried to investigate whether integrating circulating exosomal lncRNA-GC1 and the current AJCC stage system could improve the prognostic predictive ability. As shown by the time-dependent ROC curves, the 3-year and 5-year AUCs of circulating exosomal lncRNA-GC1 were all lower than those of the AJCC stage system. However, the combination of circulating exosomal lncRNA-GC1 and the AJCC stage system showed better prognostic accuracy for disease-free and overall survival than the AJCC stage system, circulating exosomal lncRNA-GC1 or any other clinicopathological characteristic alone (eFigure 13 and eTable 6). These results suggest that circulating exosomal lncRNA-GC1 may complement the AJCC stage system in the prognostic prediction.

**Figure 3:**
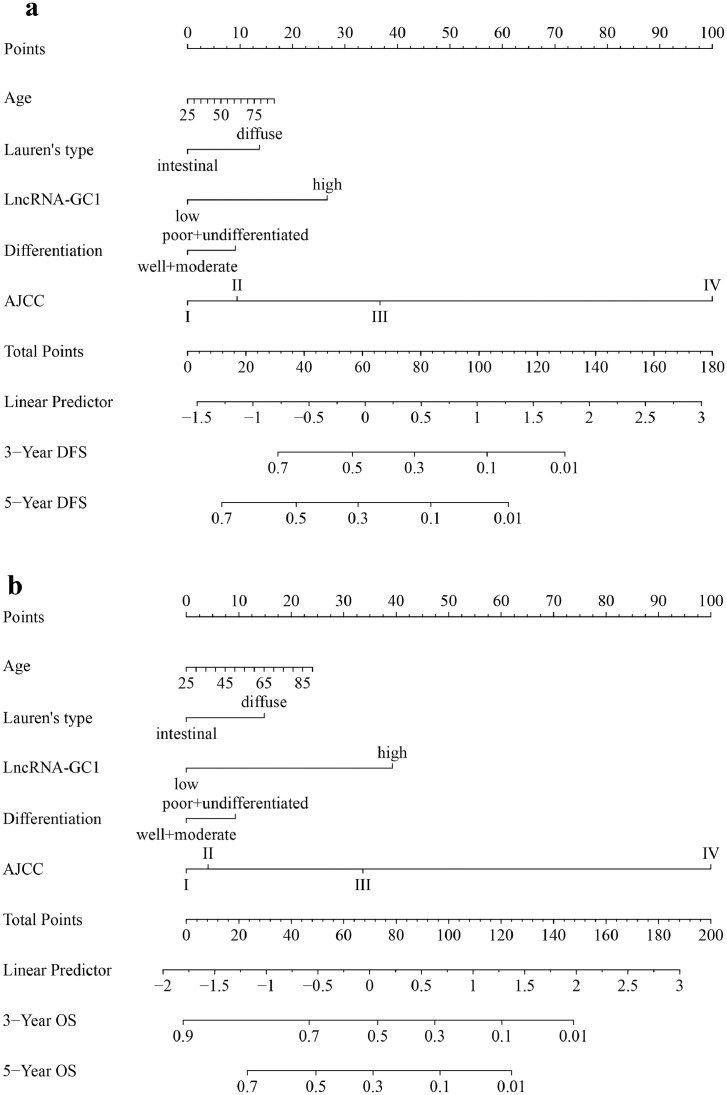
**Integrated nomograms to predict 3- and 5-year survival for patients with gastric cancer.** (a) DFS, disease-free survival; (b) OS, overall survival. To determine how many points toward the probability of DFS and OS the patient receives for circulating exosomal lncRNA-GC1, locate on the lncRNA-GC1 axis, draw a line straight upward to the point axis, repeat this process for each variable, sum the points achieved for each of the risk factors, locate the final sum on the Total Point axis, and draw a line straight down to find the patient's probability of DFS and OS.

Then, we constructed a multivariable Cox model based on independent prognostic risk factors and several known clinical prognostic factors to establish a more effective prognostic model in the training cohort.^28^ Included covariates were age, AJCC stage, circulating exosomal lncRNA-GC1, Lauren's type, differentiation status. The test of Cox proportional hazards assumption showed that each covariates test and global test were P > 0.05, which means the multivariable Cox model did not violate the assumption of proportional hazard (eFigure 14 and eTable 7). Thus, a nomogram incorporating age, differentiation status, Lauren's type, circulating exosomal lncRNA-GC1 and the AJCC stage was developed (Figure 3).

The discrimination of the nomogram was compared with those of AJCC stage system and the model2 which only incorporating age, differentiation status, Lauren's type and the AJCC stage but excluding circulating exosomal lncRNA-GC1. In the training cohort, the results showed that the C-index of the nomogram (DFS: 0.701; OS: 0.720,) was significantly higher than that of model2 (DFS: 0.669; OS: 0.675,) and the AJCC stage system (DFS: 0.645; OS: 0.648) (Both P<0.05) (eTable 8). The AUCs of the nomogram (DFS: 0.660; OS: 0.706) were higher than that of the model2 (DFS: 0.626; OS: 0.633) and the AJCC stage system (DFS: 0.614; OS: 0.611) (Both P<0.05) (eFigure 15 a; eTable 8). The same result was obtained for time-dependent AUC analysis (eFigure 16 a). The calibration curves revealed good consistency between estimated and actual probabilities of prognosis in the training cohort (eFigure 17 a). Finally, in validation phase, we performed internal validation with an independent internal cohort and external validation with two cohorts from two other hospitals. The prognostic predictive power of this model remained stable with highest C-index and AUC (eTable 8), and well-performed time-dependent AUC curves and calibration curves (eFigure 16 c-d; eFigure 17 c-d) for validation cohorts. These results suggest that this newly developed model based on circulating exosomal lncRNA-GC1 showed promising discrimination and calibration for predicting individualized prognosis of patients with GC.

### Predictive value of circulating exosomal lncRNA-GC1 for chemotherapy response

**Figure 4:**
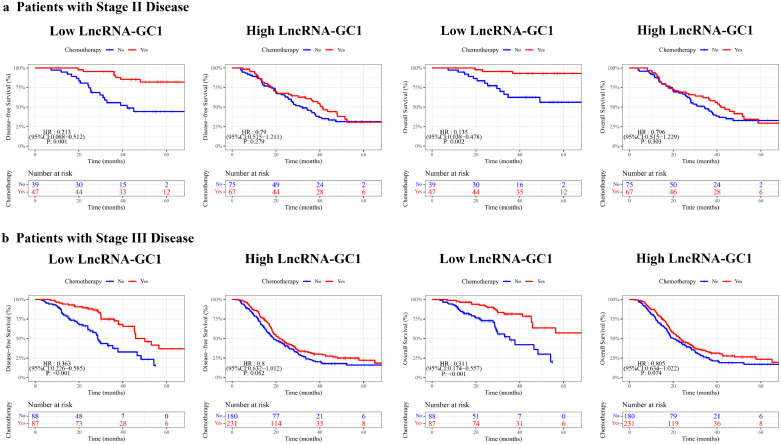
**Associations between circulating exosomal lncRNA-GC1 and survival benefit from fluorouracil-based adjuvant chemotherapy in patients with advanced gastric cancer.** In either AJCC stage II (a) or III (b) gastric cancer, fluorouracil-based adjuvant chemotherapy was significant correlated with superior disease-free survival (DFS) and overall survival (OS) in patients with low-level of circulating exosomal lncRNA-GC1, whereas no effect on survival was detected in patients with high-level of circulating exosomal lncRNA-GC1.

In the last interaction phase, we investigated the correlation between circulating exosomal lncRNA-GC1 and chemotherapy benefit by multivariable Cox model. An interaction test showed that patients with either stage II or III GC, benefited more from adjuvant chemotherapy among patients with low-level lncRNA-GC1 than those with high-level (DFS: stage II: HR, 0.213; 95%CI, 0.088–0.512; P=0.001; stage III: HR, 0.363; 95%CI, 0.226-0.585; P<0.001; both P < 0.05 for interaction; OS: stage II: HR, 0.135; 95%CI, 0.038–0.478; P=0.002; stage III: HR, 0.311; 95%CI, 0.174–0.557; P<0.001; both P < 0.05 for interaction) (eTable 9). The corresponding Kaplan–Meier survival curves for patients with stage II or stage III GC, which comprehensively compared low-level with high-level lncRNA-GC1 by treatment, are shown in Figure 4. Hence, these results suggest that stage II and III GC patients with low-level circulating exosomal lncRNA-GC1 may benefit more from fluorouracil-based adjuvant chemotherapy.

## Discussion

Gastric cancer is a highly heterogeneous disease with large variations in clinical outcomes, even underwent same postoperative chemotherapy.^1^, ^2^, ^3^ Thus, accurate prediction of prognosis and treatment response is of great importance to determine which treatment approach patients may benefit more from. To address this formidable challenge, we conducted this multi-cohort, multi-phase study. In discovery phase, we identified circulating exosomal lncRNA-GC1 as an independent prognostic factor for patients with gastric cancer and developed a predictive model based on this new molecular feature and the traditional AJCC stage system with promising efficiency for predicting individualized prognosis. In validation phase, this model remained its predictive value in three independent cohorts. In interaction phase, circulating exosomal lncRNA-GC1 has the potential to select patients who will benefit more from chemotherapy.

Currently, there are no reliable serum biomarkers used for predicting prognosis and treatment response in clinical practice. Although traditional biomarkers CEA and CA19-9 may play early warning roles in a small number of patients with GC, the overall specificity and sensitivity are still inadequate to meet clinical needs.^31^, ^32^, ^33^ With the advent of high-throughput proteogenomic characterization, more and more studies have identified subtypes of GC and gained popularity in guiding treatment and prognosis.^34^, ^35^, ^36^ Under this background, for the first time, we systematically identified the predictive value of circulating exosomal lncRNA-GC1 for prognosis and treatment response. Circulating exosomes secreted by tumor cells, have the unique advantages of not only being an accurate and noninvasive reflection of tumor cells, but also can be detected repeatedly throughout the treatment course.^22^^,^^37^ Circulating exosomal lncRNA-GC1 analysis may reveal subtle gastric cancer phenotypes that reflect the sensitivity of tumor cells to chemotherapy regimens. Indeed, several studies have attempted to develop biomarkers for predicting treatment response. Jiang et al. has developed a radiomics signatures that could predict response to immunotherapy.^38^ Cao et al has investigated that O6-methylguanine-DNA methyltransferase (MGMT) protein expression loss serves as a favorable prognostic factor and the model based on MGMT lead to better predictive accuracy.^39^ In another study, Zhang et al correlated tumor-infiltrating neutrophils with superior survival and generated a postoperative risk stratification model.^40^ To our knowledge, these studies are proof-of-principle investigations with a relatively small number of participants and lacking of external validation. Here, our model based on circulating exosomal lncRNA-GC1 was developed and validated in a relatively large number of 981 patients from three independent medical centers. Moreover, these three hospitals have large number and relatively broad geographical distribution of GC patients, each of them can be representative of the wider population in our country. Although there is a long way for further validation of this model, we envision that this predictive model will be helpful for surgeons to facilitate decision making in treatment choices.

In Asian countries, for patients with advanced gastric cancer, the standard treatment approach is adjuvant chemotherapy after surgery to prevent recurrence and improve survival.^5^^,^^41^, ^42^, ^43^ This postoperative treatment choice is largely based on two pivotal Phase III trials, the ACTS-GC and the CLASSSIC, which have demonstrated the efficacy of fluorouracil-based adjuvant chemotherapy after surgery.^43^^,^^44^ However, the 5-year survival analysis of the CLASSSIC trial indicated that patients in adjuvant-chemotherapy group only had a slightly increased overall survival rate than those in surgery-only group (78% vs. 69%). These findings strongly highlight that many patients may not benefit from adjuvant chemotherapy given the excessive toxicity for responders and delayed assess to an alternative treatment for non-responders. For now, there is no optimal criteria for screening potential candidates who may benefit from chemotherapy. In this study, we showed that circulating exosomal lncRNA-GC1 was able to distinguish patients who are suitable for chemotherapy. Specifically, patients with low circulating exosomal lncRNA-GC1 are more likely to derive disease-free and overall survival benefit from adjuvant chemotherapy. Mechanically, lncRNA-GC1 functions as an oncogenic scaffold to facilitate histone modification and therefore promote several proteins transcription which may induce chemotherapy resistance.^23^ Thus, the high-level of lncRNA-GC1 in GC cells and exosomes may be reasonable to show inherently insensitive to fluorouracil-based chemotherapy. Thus, we hold the opinion that patients with high-level of circulating exosomal lncRNA-GC1 may not benefit from fluorouracil-based chemotherapy and should treated with other regimens. Even so, future trials are still needed to integrate lncRNA-GC1 signatures to personalize the postoperative treatment.

In western countries, neoadjuvant chemotherapy is typically the first choice for patients with advanced gastric cancer, which is largely based on two Phase III trials, the MAGIC and the FNCLCC/FFCD. These two trials suggest the advantages of neoadjuvant chemotherapy including a high rate of R0 resection and the avoidance of unnecessary surgery.^6^^,^^42^ Although our study is focused on postoperative adjuvant chemotherapy, it is possible that this model may also be applicable to neoadjuvant chemotherapy. The main reason is that our model is developed with pretreatment circulating exosomal lncRNA-GC1 and accurately reflects the underlying biological characteristics of tumors independent of treatment. Besides, the high-level of lncRNA-GC1 in tumor cells and exosomes may indicate the inherent insensitivity to chemotherapy.^23^ Thus, we envision that a noninvasive prediction for prognosis and treatment response may be valuable with the possibility of early assessment prior to surgery, and this will be needed to be identified in our future work.

In our opinion, there is a pivotal strength of this study. Based on this predictive model, we enrolled not only the routine clinicopathological characteristics, but also the specific molecular feature of lncRNA-GC1. These findings are motivated by the fact that circulating exosomal lncRNA-GC1 contributes both to the chemotherapy resistance mechanisms and valuable prognostic information. Hence, this predictive model is a comprehensive reflection of gastric cancer and provides adequate information for surgeons in clinical routines.

Although circulating exosomal lncRNA-GC1 showed promising predictive value, this study has several limitations. The primary limitation is the retrospective nature. To address this, we systemically conducted this multi-phase study with four independent cohorts from three different medical centers. We aimed to ensure reliability and reproducibility with patients from different cohorts. Furthermore, to avoid susceptible to potential selection bias, whether patients receive adjuvant chemotherapy after surgery or not was determined by the multidisciplinary team. Importantly, we give great emphasis on the generalizability of the model across different cohorts from different medical centers, and the efficiency remains stable. In future work, the ultimate validation is through prospective, randomized trials in diverse populations.

In conclusion, to the best of our knowledge, this is the first study to comprehensively evaluate the predictive value of circulating exosomal lncRNA-GC1 for individualized prognosis. The most profound findings of this study are that circulating exosomal lncRNA-GC1 have the potential to predict chemotherapy response and select patients who would benefit more from fluorouracil-based chemotherapy. The measurement of circulating exosomal lncRNA-GC1 may complement the currently standard AJCC stage-based assessment to provide a more accurate prognostic prediction and treatment choice selection for patients with advanced gastric cancer. This novel biomarker cannot be widely used to guide clinical practice before the identification in a randomized trial, however, this study may shed new lights as the first step to achieve this long-term goal.

### Contributors

Xinxin Wang and Xin Guo had verified the underlying data and had full access to all the data in the study and take responsibility for the integrity of the data and the accuracy of the data analysis. Xiaohui Lv and Xin Guo were associated with Funding acquisition. Xinxin Wang, Xin Guo and Qiying Song designed the study; Xiaohui Lv, Yi Ru and Jian Dong performed the experiments; Qiying Song, Di Wu, Rongyan Chang and Lubin Chen collected the samples of individuals; Xiaohui Lv and Qiying Song performed statistical analysis; Xin Guo wrote the paper; Qiying Song, Xiaohui Lv, Xinxin Wang and Xin Guo revised the manuscript and were responsible for the decision to submit the manuscript. All the authors read and approved the submitted manuscript.

### Data sharing statement

The data and materials of the study will be made available on request.

## Declaration of interests

All authors declare no potential conflict of interest.

## Appendix Supplementary materials

Image, application 1

Image, application 2

Image, application 3

Image, application 4

Image, application 5

Image, application 6

Image, application 7

Image, application 8

Image, application 9

Image, application 10
