# Supplementary material for: Circulating exosomal gastric cancer-associated long noncoding RNA1 as a noninvasive biomarker for predicting chemotherapy response and prognosis of advanced gastric cancer: A multi-cohort, multi-phase study
Source: eBioMedicine. 2022 Mar 27;78:103971. doi: 10.1016/j.ebiom.2022.103971 (PMC8965144; doi:10.1016/j.ebiom.2022.103971)
Supplement: Supplementary file 1 [file mmc1.docx]

**eFigures**

**eFigure.1** **ROC curve of circulating exosomal lncRNA-GC1 in training cohort in the discovery phases.**

**eFigure.2 Kaplan-Meier analyses of DFS and OS according to the levels of circulating exosomal lncRNA-GC1 in patients with gastric cancer in different cohorts.**

(a) Internal validation cohort (n=262); (b) External validation cohort 1 (n=186); (c) External validation cohort 2 (n=158); (d) Combined cohort (n=981). left panel: DFS; right panel: OS. DFS, disease-free survival. OS, overall survival. HR, hazard ratio. CI, confidence interval.

**eFigure.3 Kaplan-Meier analyses of OS according to the levels of circulating exosomal lncRNA-GC1 and AJCC stage in different cohorts.**

(a) Training cohort (n=375); (b) Internal validation cohort (n=262); (c) External validation cohort 1 (n=186); (d) External validation cohort 2 (n=158). left panel: AJCC stage I; middle panel: AJCC stage II; right panel: AJCC stage III. DFS, disease-free survival. HR, hazard ratio.

**eFigure.4. Kaplan-Meier analyses of DFS according to the levels of circulating exosomal lncRNA-GC1 and AJCC stage in different cohorts.**

(a) Training cohort (n=375); (b) Internal validation cohort (n=262); (c) External validation cohort 1 (n=186); (d) External validation cohort 2 (n=158). left panel: AJCC stage I; middle panel: AJCC stage II; right panel: AJCC stage III. OS, overall survival. HR, hazard ratio.

**eFigure.5. Kaplan-Meier survival analyses of DFS for patients in training cohort according to circulating exosomal lncRNA-GC1 stratified by clinicopathological risk factors.**

**eFigure.6. Kaplan-Meier survival analyses of OS for patients in training cohort according to circulating exosomal lncRNA-GC1 stratified by clinicopathological risk factors.**

**eFigure.7. Kaplan-Meier survival analyses of DFS for patients in internal validation cohort according to circulating exosomal lncRNA-GC1 stratified by clinicopathological risk factors.**

**eFigure.8. Kaplan-Meier survival analyses of OS for patients in internal validation cohort according to circulating exosomal lncRNA-GC1 stratified by clinicopathological risk factors.**

**eFigure.9. Kaplan-Meier survival analyses of DFS for patients in external validation cohort 1 according to circulating exosomal lncRNA-GC1 stratified by clinicopathological risk factors.**

**eFigure.10. Kaplan-Meier survival analyses of OS for patients in external validation cohort 1 according to circulating exosomal lncRNA-GC1 stratified by clinicopathological risk factors.**

**eFigure.11. Kaplan-Meier survival analyses of DFS for patients in external validation cohort 2 according to circulating exosomal lncRNA-GC1 stratified by clinicopathological risk factors.**

**eFigure.12. Kaplan-Meier survival analyses of OS for patients in external validation cohort 2 according to circulating exosomal lncRNA-GC1 stratified by clinicopathological risk factors.**

**eFigure.13. Time-dependent ROC curves for assessing circulating exosomal lncRNA-GC1, AJCC stage and clinicopathological characteristics as predictors of disease-free and overall survival in different cohorts.**

(a) Training cohort (n=375); (b) Internal validation cohort (n=262); (c) External validation cohort 1 (n=186); (d) External validation cohort 2 (n=158). ROC, receiver operating characteristic.

**eFigure.14. Tests of proportional Hazards assumption in the training cohort.**

(a): DFS; (b): OS. DFS.

**eFigure.15**. **The ROC curves compared the predictive value of three models in different cohort.**

Model1: Nomogram (Age, Differentiation status, Lauren type, Circulating exosomal lncRNA-GC1 and the AJCC stage); Model2: Age, Differentiation status, Lauren type and the AJCC stage; Model3: AJCC stage system. Left panel: DFS; Right panel: OS.

**eFigure.16. Time-dependent AUC curves to show prognostic power of three models as predictors of disease-free and overall survival in different cohorts.**

(a) Training cohort (n=375); (b) Internal validation cohort (n=262); (c) External validation cohort 1 (n=186); (d) External validation cohort 2 (n=158). Left panel: DFS; Right panel: OS.

Model1: Nomogram (Age, Differentiation status, Lauren type, Circulating exosomal lncRNA-GC1 and the AJCC stage); Model2: Age, Differentiation status, Lauren type and the AJCC stage; Model3: AJCC stage system

Time-dependent area under the ROC curve (AUC) analyses shows the prognostic capacity of three models. Highest prognostic power is also observed in the nomogram. Horizontal axis represents survival time (Months). Vertical axis represents area under the ROC curve (AUC).

**eFigure.17. The calibration curves for assessing performance of the nomogram.**

(a) Training cohort (n=375); (b) Internal validation cohort (n=262); (c) External validation cohort 1 (n=186); (d) External validation cohort 2 (n=158).

The x‑axis represents the nomogram‑predicted survival, and the y‑axis represents the observed survival. The line represents the ideal reference line where predicted survival corresponds with the observed survival.

**eTables**

**eTable.1**. Correlations between circulating exosomal lncRNA-GC1 and clinicopathological characteristics of patients in training and validation cohorts.

**eTable.2**. Univariable and multivariable analysis of patients in training cohort.

**eTable.3**. Univariable and multivariable analysis of patients in internal validation cohort.

**eTable.4.** Univariable and multivariable analysis of patients in external validation cohort 1.

**eTable.5**. Univariable and multivariable analysis of patients in external validation cohort 2.

**eTable.6**. Time-dependent ROC curves for circulating exosomal lncRNA-GC1, AJCC stage and clinicopathological characteristics as predictors of disease-free and overall survival in the training cohort and validation cohorts.

**eTable.7**. Tests of proportional Hazards assumption in the training cohort.

**eTable.8.** Comparing the C-index and AUC of three models in the training and validation cohorts.

**eTable.9**. Treatment interaction with circulating exosomal lncRNA-GC1 for disease-free and overall survival.
