# Supplementary material for: Circulating exosomal gastric cancer-associated long noncoding RNA1 as a noninvasive biomarker for predicting chemotherapy response and prognosis of advanced gastric cancer: A multi-cohort, multi-phase study
Source: eBioMedicine. 2022 Mar 27;78:103971. doi: 10.1016/j.ebiom.2022.103971 (PMC8965144; doi:10.1016/j.ebiom.2022.103971)
Supplement: Supplementary file 2 [file mmc2.docx]

**eTable.1.** **Correlations between circulating exosomal lncRNA-GC1 and clinicopathological characteristics of patients in training and validation cohorts**

| **Factors** | **Training cohort** | | | **Internal validation cohort** | | | **External validation cohort 1** | | | **External validation cohort 2** | | |
| --- | --- | --- | --- | --- | --- | --- | --- | --- | --- | --- | --- | --- |
|  | **Low** | **High** | **P** | **Low** | **High** | **P** | **Low** | **High** | **P** | **Low** | **High** | **P** |
| All patients | 144 | 231 |  | 91 | 171 |  | 66 | 120 |  | 59 | 99 |  |
| Age (years) |  |  | 0.485 |  |  | 0.568 |  |  | 0.158 |  |  | 0.950 |
| ≤60 | 77 | 150 |  | 58 | 112 |  | 31 | 69 |  | 28 | 59 |  |
| >60 | 46 | 102 |  | 42 | 50 |  | 20 | 66 |  | 26 | 45 |  |
| Gender |  |  | 0.616 |  |  | 0.551 |  |  | 0.174 |  |  | 0.840 |
| Male | 101 | 155 |  | 56 | 113 |  | 36 | 79 |  | 37 | 65 |  |
| Female | 43 | 76 |  | 35 | 58 |  | 30 | 41 |  | 22 | 34 |  |
| Tumor location |  |  | 0.546 |  |  | 0.596 |  |  | 0.124 |  |  | 0.240 |
| Cardia | 34 | 41 |  | 20 | 38 |  | 15 | 28 |  | 7 | 19 |  |
| Body | 22 | 39 |  | 11 | 22 |  | 9 | 21 |  | 8 | 15 |  |
| Antrum | 71 | 118 |  | 42 | 88 |  | 39 | 54 |  | 31 | 54 |  |
| Whole | 17 | 33 |  | 18 | 23 |  | 3 | 17 |  | 13 | 11 |  |
| Differentiation status |  |  | 0.711 |  |  | 0.167 |  |  | 0.298 |  |  | 0.777 |
| Well + moderate | 47 | 81 |  | 31 | 43 |  | 22 | 30 |  | 17 | 32 |  |
| Poor + undifferentiated | 97 | 150 |  | 60 | 128 |  | 44 | 90 |  | 42 | 67 |  |
| Lauren type |  |  | 0.894 |  |  | 0.923 |  |  | 0.969 |  |  | 0.611 |
| Intestinal | 103 | 168 |  | 69 | 132 |  | 48 | 89 |  | 43 | 67 |  |
| Diffuse or mixed | 41 | 63 |  | 22 | 39 |  | 18 | 31 |  | 16 | 32 |  |
| Depth of invasion |  |  | **<0.001** |  |  | **0.001** |  |  | **0.020** |  |  | **0.047** |
| pT1 | 27 | 14 |  | 13 | 5 |  | 9 | 7 |  | 9 | 8 |  |
| pT2 | 16 | 10 |  | 8 | 12 |  | 8 | 4 |  | 7 | 2 |  |
| pT3 | 47 | 83 |  | 38 | 62 |  | 23 | 38 |  | 20 | 41 |  |
| pT4 | 54 | 124 |  | 32 | 92 |  | 26 | 71 |  | 23 | 48 |  |
| Lymph node metastasis |  |  | **0.022** |  |  | **0.045** |  |  | **0.042** |  |  | **0.028** |
| pN0 | 60 | 73 |  | 35 | 46 |  | 29 | 29 |  | 23 | 25 |  |
| pN1 | 25 | 27 |  | 18 | 22 |  | 11 | 16 |  | 17 | 16 |  |
| pN2 | 31 | 60 |  | 21 | 44 |  | 12 | 35 |  | 8 | 29 |  |
| pN3 | 28 | 71 |  | 17 | 59 |  | 14 | 40 |  | 11 | 29 |  |
| Metastasis |  |  | 0.768 |  |  | 0.612 |  |  | 0.240 |  |  | 1.000 |
| M0 | 138 | 224 |  | 90 | 166 |  | 64 | 120 |  | 57 | 95 |  |
| M1 | 6 | 7 |  | 1 | 5 |  | 2 | 0 |  | 2 | 4 |  |
| AJCC stage |  |  | **<0.001** |  |  | **0.002** |  |  | **0.001** |  |  | **0.045** |
| I | 37 | 21 |  | 21 | 13 |  | 16 | 10 |  | 14 | 18 |  |
| II | 32 | 59 |  | 22 | 34 |  | 17 | 24 |  | 15 | 25 |  |
| III | 69 | 144 |  | 47 | 119 |  | 31 | 86 |  | 28 | 62 |  |
| IV | 6 | 7 |  | 1 | 5 |  | 2 | 0 |  | 2 | 4 |  |
| Chemotherapy^a^ |  |  | 0.396 |  |  | 0.205 |  |  | 0.392 |  |  | 0.712 |
| Yes | 70 | 124 |  | 37 | 85 |  | 29 | 62 |  | 29 | 53 |  |
| No | 74 | 107 |  | 54 | 86 |  | 37 | 58 |  | 30 | 46 |  |

^a^ Patients with GC received fluorouracil-based chemotherapy for at least 4 cycles.
