# Supplementary material for: Circulating exosomal gastric cancer-associated long noncoding RNA1 as a noninvasive biomarker for predicting chemotherapy response and prognosis of advanced gastric cancer: A multi-cohort, multi-phase study
Source: eBioMedicine. 2022 Mar 27;78:103971. doi: 10.1016/j.ebiom.2022.103971 (PMC8965144; doi:10.1016/j.ebiom.2022.103971)
Supplement: Supplementary file 3 [file mmc3.docx]

**eTable.2. Univariable and multivariable analysis of patients in training cohort**

| **Factors** |  | **Disease-free survival** | | **Overall survival** | |
| --- | --- | --- | --- | --- | --- |
|  |  | **HR (95% CI)** | ***P* value** | **HR (95% CI)** | ***P* value** |
| Univariable analysis |  |  |  |  |  |
| **Circulating exosomal lncRNA-GC1** | Low | 1.000 (Reference) | **<0.001** | 1.000 (Reference) | **<0.001** |
|  | High | 2.159 (1.602-2.911) |  | 2.834 (2.038-3.940) |  |
| Gender | Male | 1.000 (Reference) | 0.334 | 1.000 (Reference) | 0.347 |
|  | Female | 1.152 (0.865-1.535) |  | 1.154 (0.857-1.554) |  |
| Age (years) | ≤60 | 1.000 (Reference) | **0.027** | 1.000 (Reference) | **0.007** |
|  | >60 | 1.014 (1.002-1.026) |  | 1.017 (1.005-1.030) |  |
| Tumor location | Cardia | 1.000 (Reference) | 0.575 | 1.000 (Reference) | 0.579 |
|  | Body | 1.079 (0.679-1.714) |  | 1.074 (0.667-1.729) |  |
|  | Antrum | 1.019 (0.703-1.477) |  | 0.975 (0.665-1.431) |  |
|  | Whole | 1.344 (0.835-2.161) |  | 1.305 (0.800-2.130) |  |
| Differentiation status | Well + moderate | 1.000 (Reference) | 0.094 | 1.000 (Reference) | 0.103 |
|  | Poor + undifferentiated | 1.289 (0.957-1.736) |  | 1.294 (0.949-1.764) |  |
| Lauren type | Intestinal | 1.000 (Reference) | 0.058 | 1.000 (Reference) | 0.092 |
|  | Diffuse or mixed | 1.340 (0.990-1.815) |  | 1.312 (0.956-1.800) |  |
| AJCC stage | I | 1.000 (Reference) | **<0.001** | 1.000 (Reference) | **<0.001** |
|  | II | 1.642 (0.983-2.742) |  | 1.554 (0.913-2.645) |  |
|  | III | 3.238 (2.047-5.124) |  | 3.211 (2.003-5.147) |  |
|  | IV | 17.211 (8.457-35.029) |  | 16.359 (7.983-33.523) |  |
| Multivariable analysis |  |  |  |  |  |
| **Circulating exosomal lncRNA-GC1** | Low | 1.000 (Reference) | **<0.001** | 1.000 (Reference) | **<0.001** |
|  | High | 1.972 (1.453-2.676) |  | 2.659 (1.898-3.724) |  |
| Age (≤60 vs. >60) | ≤60 | 1.000 (Reference) | 0.417 | 1.000 (Reference) | 0.237 |
|  | >60 | 1.005 (0.993-1.017) |  | 1.007 (0.995-1.020) |  |
| AJCC stage | I | 1.000 (Reference) | **<0.001** | 1.000 (Reference) | **<0.001** |
|  | II | 1.312 (0.778-2.212) |  | 1.146 (0.667-1.969) |  |
|  | III | 2.611 (1.635-4.172) |  | 2.376 (1.468-3.845) |  |
|  | IV | 14.968 (7.229-30.992) |  | 14.150 (6.761-29.615) |  |
