# Supplementary material for: Circulating exosomal gastric cancer-associated long noncoding RNA1 as a noninvasive biomarker for predicting chemotherapy response and prognosis of advanced gastric cancer: A multi-cohort, multi-phase study
Source: eBioMedicine. 2022 Mar 27;78:103971. doi: 10.1016/j.ebiom.2022.103971 (PMC8965144; doi:10.1016/j.ebiom.2022.103971)
Supplement: Supplementary file 4 [file mmc4.docx]

**eTable.3. Univariable and multivariable analysis of patients in internal validation cohort**

| **Factors** |  | **Disease-free survival** | | **Overall survival** | |
| --- | --- | --- | --- | --- | --- |
|  |  | **HR (95% CI)** | ***P* value** | **HR (95% CI)** | ***P* value** |
| Univariable analysis |  |  |  |  |  |
| **Circulating exosomal lncRNA-GC1** | Low | 1.000 (Reference) | **<0.001** | 1.000 (Reference) | **<0.001** |
|  | High | 3.106 (2.102-4.589) |  | 4.750 (2.935-7.690) |  |
| Gender | Male | 1.000 (Reference) | 0.697 | 1.000 (Reference) | 0.648 |
|  | Female | 1.068 (0.767-1.487) |  | 1.086 (0.761-1.550) |  |
| Age (years) | ≤60 | 1.000 (Reference) | 0.126 | 1.000 (Reference) | 0.153 |
|  | >60 | 1.011 (0.997-1.026) |  | 1.011 (0.996-1.027) |  |
| Tumor location | Cardia | 1.000 (Reference) | 0.549 | 1.000 (Reference) | 0.738 |
|  | Body | 0.740 (0.401-1.368) |  | 0.814 (0.427-1.553) |  |
|  | Antrum | 1.110 (0.737-1.671) |  | 1.094 (0.700-1.709) |  |
|  | Whole | 1.054 (0.624-1.780) |  | 1.149 (0.658-2.006) |  |
| Differentiation status | Well + moderate | 1.000 (Reference) | 0.074 | 1.000 (Reference) | **0.022** |
|  | Poor + undifferentiated | 1.416 (0.967-2.074) |  | 1.655 (1.077-2.542) |  |
| Lauren type | Intestinal | 1.000 (Reference) | 0.059 | 1.000 (Reference) | 0.072 |
|  | Diffuse or mixed | 1.424 (0.987-2.054) |  | 1.434 (0.968-2.125) |  |
| AJCC stage | I | 1.000 (Reference) | **<0.001** | 1.000 (Reference) | **<0.001** |
|  | II | 1.989 (1.031-3.837) |  | 2.221 (1.063-4.639) |  |
|  | III | 3.522 (1.949-6.364) |  | 3.826 (1.959-7.472) |  |
|  | IV | 23.379 (8.488-64.395) |  | 25.914 (8.973-74.839) |  |
| Multivariable analysis |  |  |  |  |  |
| **Circulating exosomal lncRNA-GC1** | Low | 1.000 (Reference) | **<0.001** | 1.000 (Reference) | **<0.001** |
|  | High | 2.622 (1.763-3.901) |  | 3.973 (2.437-6.479) |  |
| AJCC stage | I | 1.000 (Reference) | **<0.001** | 1.000 (Reference) | **<0.001** |
|  | II | 1.633 (0.845-3.158) |  | 1.564 (0.741-3.299) |  |
|  | III | 2.701 (1.484-4.914) |  | 2.483 (1.256-4.909) |  |
|  | IV | 15.301 (5.514-42.463) |  | 14.232 (4.897-41.362) |  |
| Differentiation status | Well + moderate |  |  | 1.000 (Reference) | 0.185 |
|  | Poor + undifferentiated |  |  | 1.346 (0.868-2.086) |  |
