# Supplementary material for: Circulating exosomal gastric cancer-associated long noncoding RNA1 as a noninvasive biomarker for predicting chemotherapy response and prognosis of advanced gastric cancer: A multi-cohort, multi-phase study
Source: eBioMedicine. 2022 Mar 27;78:103971. doi: 10.1016/j.ebiom.2022.103971 (PMC8965144; doi:10.1016/j.ebiom.2022.103971)
Supplement: Supplementary file 5 [file mmc5.docx]

**eTable.4. Univariable and multivariable analysis of patients in external validation cohort 1**

| **Factors** |  | **Disease-free survival** | | **Overall survival** | |
| --- | --- | --- | --- | --- | --- |
|  |  | **HR (95% CI)** | ***P* value** | **HR (95% CI)** | ***P* value** |
| Univariable analysis |  |  |  |  |  |
| **Circulating exosomal lncRNA-GC1** | Low | 1.000 (Reference) | **<0.001** | 1.000 (Reference) | **<0.001** |
|  | High | 3.923 (2.245-6.854) |  | 3.928 (2.248-6.863) |  |
| Gender | Male | 1.000 (Reference) | 0.691 | 1.000 (Reference) | 0.700 |
|  | Female | 0.917 (0.597-1.408) |  | 0.919 (0.598-1.412) |  |
| Age (years) | ≤60 | 1.000 (Reference) | **0.009** | 1.000 (Reference) | **0.009** |
|  | >60 | 1.732 (1.148-2.614) |  | 1.733 (1.149-2.615) |  |
| Tumor location | Cardia | 1.000 (Reference) | **0.026** | 1.000 (Reference) | **0.027** |
|  | Body | 1.258 (0.619-2.555) |  | 1.255 (0.618-2.55) |  |
|  | Antrum | 1.238 (0.71-2.16) |  | 1.226 (0.703-2.14) |  |
|  | Whole | 2.726 (1.354-5.487) |  | 2.71 (1.347-5.453) |  |
| Differentiation status | Well + moderate | 1.000 (Reference) | **0.009** | 1.000 (Reference) | **0.008** |
|  | Poor + undifferentiated | 1.997 (1.191-3.349) |  | 2.002 (1.194-3.358) |  |
| Lauren type | Intestinal | 1.000 (Reference) | 0.613 | 1.000 (Reference) | 0.613 |
|  | Diffuse or mixed | 1.128 (0.707-1.8) |  | 1.128 (0.707-1.801) |  |
| AJCC stage | I | 1.000 (Reference) | **0.001** | 1.000 (Reference) | **0.001** |
|  | II | 1.562 (0.689-3.543) |  | 1.561 (0.688-3.54) |  |
|  | III | 3.135 (1.523-6.455) |  | 3.133 (1.522-6.451) |  |
|  | IV | 16.151 (1.925-135.501) |  | 16.825 (1.999-141.613) |  |
| Multivariable analysis |  |  |  |  |  |
| **Circulating exosomal lncRNA-GC1** | Low | 1.000 (Reference) | **<0.001** | 1.000 (Reference) | **<0.001** |
|  | High | 3.228(1.785 - 5.838) |  | 3.216(1.777 - 5.819) |  |
| Age (years) | ≤60 | 1.000 (Reference) | **0.041** | 1.000 (Reference) | **0.041** |
|  | >60 | 1.552(1.019 - 2.363) |  | 1.552(1.019 - 2.365) |  |
| Tumor location | Cardia | 1.000 (Reference) | 0.762 | 1.000 (Reference) | 0.785 |
|  | Body | 1.147(0.561 - 2.341) |  | 1.145(0.56 - 2.338) |  |
|  | Antrum | 1.119(0.631 - 1.986) |  | 1.098(0.618 - 1.95) |  |
|  | Whole | 1.446(0.699 - 2.989) |  | 1.428(0.691 - 2.954) |  |
| Differentiation status | Well + moderate | 1.000 (Reference) | **0.032** | 1.000 (Reference) | **0.032** |
|  | Poor + undifferentiated | 1.78(1.05 - 3.016) |  | 1.783(1.052 - 3.023) |  |
| AJCC stage | I | 1.000 (Reference) | **0.017** | 1.000 (Reference) | **0.014** |
|  | II | 1.229(0.54 - 2.799) |  | 1.227(0.539 - 2.795) |  |
|  | III | 1.959(0.942 - 4.078) |  | 1.954(0.938 - 4.069) |  |
|  | IV | 22.401(2.496-201.092) |  | 23.238(2.584- 209.002) |  |
