# Supplementary material for: Circulating exosomal gastric cancer-associated long noncoding RNA1 as a noninvasive biomarker for predicting chemotherapy response and prognosis of advanced gastric cancer: A multi-cohort, multi-phase study
Source: eBioMedicine. 2022 Mar 27;78:103971. doi: 10.1016/j.ebiom.2022.103971 (PMC8965144; doi:10.1016/j.ebiom.2022.103971)
Supplement: Supplementary file 6 [file mmc6.docx]

**eTable.5. Univariable and multivariable analysis of patients in external validation cohort 2**

| **Factors** |  | **Disease-free survival** | | **Overall survival** | |
| --- | --- | --- | --- | --- | --- |
|  |  | **HR (95% CI)** | ***P* value** | **HR (95% CI)** | ***P* value** |
| Univariable analysis |  |  |  |  |  |
| **Circulating exosomal lncRNA-GC1** | Low | 1.000 (Reference) | **<0.001** | 1.000 (Reference) | **<0.001** |
|  | High | 2.961 (1.781-4.923) |  | 4.012 (2.209-7.287) |  |
| Gender | Male | 1.000 (Reference) | 0.908 | 1.000 (Reference) | 0.589 |
|  | Female | 1.026 (0.661-1.592) |  | 0.877 (0.543-1.414) |  |
| Age (years) | ≤60 | 1.000 (Reference) | **0.002** | 1.000 (Reference) | **0.005** |
|  | >60 | 1.967 (1.291-2.996) |  | 1.91 (1.218-2.995) |  |
| Tumor location | Cardia | 1.000 (Reference) | 0.821 | 1.000 (Reference) | 0.950 |
|  | Body | 0.877 (0.421-1.829) |  | 0.791 (0.341-1.833) |  |
|  | Antrum | 0.785 (0.454-1.356) |  | 0.945 (0.516-1.732) |  |
|  | Whole | 0.938 (0.459-1.92) |  | 1.094 (0.505-2.37) |  |
| Differentiation status | Well + moderate | 1.000 (Reference) | **0.025** | 1.000 (Reference) | 0.147 |
|  | Poor + undifferentiated | 1.767 (1.074-2.907) |  | 1.456 (0.876-2.42) |  |
| Lauren type | Intestinal | 1.000 (Reference) | 0.131 | 1.000 (Reference) | 0.211 |
|  | Diffuse or mixed | 1.41 (0.903-2.201) |  | 1.357 (0.842-2.188) |  |
| AJCC stage | I | 1.000 (Reference) | **<0.001** | 1.000 (Reference) | **<0.001** |
|  | II | 4.321 (1.464-12.753) |  | 3.573 (1.19-10.73) |  |
|  | III | 8.268 (2.947-23.201) |  | 6.608 (2.335-18.699) |  |
|  | IV | 15.594 (3.786-64.235) |  | 14.125 (3.42-58.334) |  |
| Multivariable analysis |  |  |  |  |  |
| **Circulating exosomal lncRNA-GC1** | Low | 1.000 (Reference) | **<0.001** | 1.000 (Reference) | **<0.001** |
|  | High | 2.541(1.521 - 4.247) |  | 3.39(1.859 - 6.185) |  |
| Age (years) | ≤60 | 1.000 (Reference) | 0.068 | 1.000 (Reference) | **0.039** |
|  | >60 | 1.521(0.97 - 2.384) |  | 1.632(1.025 - 2.597) |  |
| AJCC stage | I | 1.000 (Reference) | **0.004** | 1.000 (Reference) | **0.012** |
|  | II | 3.571(1.201 - 10.619) |  | 2.79(0.921 - 8.449) |  |
|  | III | 5.757(2.026 - 16.354) |  | 4.486(1.569 - 12.827) |  |
|  | IV | 7.978(1.872 - 34.002) |  | 7.657(1.8 - 32.569) |  |
| Differentiation status | Well + moderate | 1.000 (Reference) | 0.081 |  |  |
|  | Poor + undifferentiated | 1.587(0.945 - 2.665) |  |  |  |
