# Supplementary material for: Circulating exosomal gastric cancer-associated long noncoding RNA1 as a noninvasive biomarker for predicting chemotherapy response and prognosis of advanced gastric cancer: A multi-cohort, multi-phase study
Source: eBioMedicine. 2022 Mar 27;78:103971. doi: 10.1016/j.ebiom.2022.103971 (PMC8965144; doi:10.1016/j.ebiom.2022.103971)
Supplement: Supplementary file 7 [file mmc7.docx]

**eTable.6.** **Time-dependent ROC curves for circulating exosomal lncRNA-GC1, AJCC stage and clinicopathological characteristics as predictors of disease-free and overall survival in training and validation cohorts.**

| **Variables** | **Training**  **cohort** | **Internal validation**  **cohort** | **External validation cohort 1** | **External validation cohort 2** |
| --- | --- | --- | --- | --- |
|  | **AUC (95% CI)** | **AUC (95% CI)** | **AUC (95% CI)** | **AUC (95% CI)** |
| **Disease-free survival (3-year)** | | | |  |
| Circulating exosomal lncRNA-GC1 + AJCC stage | 74.76 (69.10-80.42) | 79.94 (73.57-86.31) | 74.00(66.57-81.43) | 78.07(70.37-85.77) |
| AJCC stage | 71.39 (65.73-77.05) | 75.36 (68.75-81.97) | 70.15(62.76-77.54) | 73.34(65.52-81.16) |
| Circulating exosomal lncRNA-GC1 | 62.12 (56.34-67.90) | 68.43 (61.67-75.19) | 66.55(59.61-73.49) | 66.86(59.14-74.58) |
| Age | 53.26 (47.46-59.06) | 51.20 (44.28-58.12) | 57.35(49.55-65.15) | 61.73(53.46-70.00) |
| Gender | 51.28 (45.67-56.89) | 47.21 (40.25-54.17) | 49.47(41.85-57.09) | 52.54(44.39-60.69) |
| Differentiation status | 55.06 (49.38-60.74) | 53.90 (47.45-60.35) | 60.54(53.46-67.62) | 55.33(47.61-63.05) |
| Tumor location | 55.42 (49.23-61.61) | 56.11 (48.31-63.91) | 58.92(50.63-67.21) | 48.28(39.17-57.39) |
| Lauren type | 58.26 (53.42-63.10) | 57.86 (52.55-63.17) | 56.11(49.72-62.50) | 55.96(48.34-63.58) |
| **Disease-free survival (5-year)** | | | | |
| Circulating exosomal lncRNA-GC1 + AJCC stage | 80.87 (72.81-88.93) | 80.01 (68.78-91.24) | 83.52(70.66-96.38) | 86.17(74.55-97.79) |
| AJCC stage | 76.69 (68.91-84.47) | 77.09 (65.68-88.50) | 80.41(68.53-92.29) | 84.29(72.73-95.85) |
| Circulating exosomal lncRNA-GC1 | 70.64 (62.86-78.42) | 70.06 (58.93-81.19) | 77.25(64.98-89.52) | 64.45(51.63-77.27) |
| Age | 54.45 (46.12-62.78) | 56.18 (45.85-66.51) | 64.37(51.88-76.86) | 61.38(49.23-73.53) |
| Gender | 49.24 (41.01-57.47) | 48.83 (37.32-60.34) | 54.35(41.02-67.68) | 56.16(44.79-67.53) |
| Differentiation status | 49.83 (41.70-57.96) | 55.99 (44.94-67.04) | 62.34(48.68-76.00) | 58.94(46.26-71.62) |
| Tumor location | 54.08 (45.77-62.39) | 53.40 (40.21-66.59) | 53.57(38.83-68.31) | 55.32(41.68-68.96) |
| Lauren type | 55.44 (48.89-61.99) | 57.29 (49.84-64.74) | 56.93(46.82-67.04) | 50.19(38.31-62.07) |
| **Overall survival (3-year)** | | | |  |
| Circulating exosomal lncRNA-GC1 + AJCC stage | 76.44 (70.95-81.93) | 80.71 (74.44-86.98) | 74.02(66.59-81.45) | 78.45(70.67-86.23) |
| AJCC stage | 73.12 (67.55-78.69) | 74.99 (68.33-81.65) | 70.18(62.79-77.57) | 73.05(65.07-81.03) |
| Circulating exosomal lncRNA-GC1 | 64.90 (59.26-70.54) | 70.09 (63.47-76.71) | 66.55(59.61-73.49) | 67.5(59.74-75.26) |
| Age | 53.35 (47.51-59.19) | 52.37 (45.37-59.37) | 57.32(49.52-65.12) | 61.3(52.85-69.75) |
| Gender | 50.95 (45.34-56.56) | 46.37 (39.41-53.33) | 49.49(41.87-57.11) | 48.09(39.80-56.38) |
| Differentiation status | 53.81 (48.11-59.51) | 55.15 (48.72-61.58) | 60.55(53.47-67.63) | 54.3(46.32-62.28) |
| Tumor location | 55.68 (49.37-61.99) | 55.35 (47.47-63.23) | 58.96(50.67-67.25) | 48.37(39.24-57.50) |
| Lauren type | 57.79 (52.85-62.73) | 57.03 (51.54-62.52) | 56.11(49.72-62.50) | 55.13(47.39-62.87) |
| Overall survival (5-year) | | | | |
| Circulating exosomal lncRNA-GC1 + AJCC stage | 81.82 (73.94-89.74) | 82.15 (71.31-93.04) | 83.54(70.68-96.46) | 85.9(74.53-97.33) |
| AJCC stage | 76.18 (68.30-84.10) | 77.44 (65.92-89.02) | 80.45(68.57-92.39) | 83.55(71.73-95.43) |
| Circulating exosomal lncRNA-GC1 | 73.71 (65.95-81.51) | 73.99 (62.88-85.16) | 77.25(64.98-89.58) | 67.25(54.47-80.09) |
| Age | 55.49 (47.06-63.96) | 56.79 (46.30-67.33) | 64.35(51.86-76.90) | 61.47(49.12-73.88) |
| Gender | 49.29 (40.98-57.64) | 49.46 (37.82-61.16) | 54.36(41.03-67.76) | 45.68(34.2336-57.18) |
| Differentiation status | 50.10 (41.91-58.33) | 57.14 (46.03-68.31) | 62.35(48.69-76.08) | 56.69(43.68-69.77) |
| Tumor location | 55.41 (46.88-63.98) | 57.14 (44.91-69.43) | 53.62(38.89-68.43) | 50(37.40-62.67) |
| Lauren type | 54.96 (48.39-61.56) | 57.38 (49.79-65.00) | 56.92(46.83-67.07) | 49.58(37.60-61.62) |
