# Supplementary material for: Circulating exosomal gastric cancer-associated long noncoding RNA1 as a noninvasive biomarker for predicting chemotherapy response and prognosis of advanced gastric cancer: A multi-cohort, multi-phase study
Source: eBioMedicine. 2022 Mar 27;78:103971. doi: 10.1016/j.ebiom.2022.103971 (PMC8965144; doi:10.1016/j.ebiom.2022.103971)
Supplement: Supplementary file 8 [file mmc8.docx]

**eTable.7.** **Tests of proportional Hazards assumption in the training cohorts**

| **Factors** | **Disease-free survival** | | | **Overall survival** | | |
| --- | --- | --- | --- | --- | --- | --- |
|  | **chisq** | **df** | ***P* value** | **chisq** | **df** | ***P* value** |
| Age | 0.666 | 1 | 0.414 | 0.458 | 1 | 0.499 |
| Lauren type | 0.200 | 1 | 0.655 | 0.208 | 1 | 0.649 |
| Circulating exosomal lncRNA-GC1 | 2.375 | 1 | 0.123 | 0.219 | 1 | 0.640 |
| Differentiation status | 0.395 | 1 | 0.530 | 0.079 | 1 | 0.778 |
| AJCC stage | 4.452 | 3 | 0.217 | 5.512 | 3 | 0.138 |
| Global test | 6.754 | 7 | 0.455 | 6.092 | 7 | 0.529 |
