# Supplementary material for: Circulating exosomal gastric cancer-associated long noncoding RNA1 as a noninvasive biomarker for predicting chemotherapy response and prognosis of advanced gastric cancer: A multi-cohort, multi-phase study
Source: eBioMedicine. 2022 Mar 27;78:103971. doi: 10.1016/j.ebiom.2022.103971 (PMC8965144; doi:10.1016/j.ebiom.2022.103971)
Supplement: Supplementary file 9 [file mmc9.docx]

**eTable.8.** **Comparing the C-index and AUC of three models in the training and validation cohorts.**

| Models | Disease-free Survival | | | | Overall Survival | | | |
| --- | --- | --- | --- | --- | --- | --- | --- | --- |
|  | C-index (95CI%) | P value | AUC (95%CI) ^a^ | P value | C-index (95CI%) | P value | AUC (95%CI) ^a^ | P value |
| Training cohort |  |  |  |  |  |  |  |  |
| Model1 | 0.701(0.666-0.736) |  | 0.660(0.605-0.716) |  | 0.720(0.683-0.757) |  | 0.706(0.653-0.758) |  |
| Model2 | 0.669(0.632-0.706) | 0.005 | 0.626(0.570-0.683) | <0.001 | 0.675(0.638-0.712) | 0.001 | 0.633(0.577-0.689) | <0.001 |
| Model3 | 0.645(0.612-0.678) | <0.001 | 0.614(0.563-0.664) | <0.001 | 0.648(0.615-0.681) | <0.001 | 0.611(0.561-0.662) | <0.001 |
| Internal cohort |  |  |  |  |  |  |  |  |
| Model1 | 0.692(0.651-0.733) |  | 0.708(0.644-0.771) |  | 0.719(0.678-0.760) |  | 0.755(0.697-0.814) |  |
| Model2 | 0.634(0.589-0.679) | <0.001 | 0.631(0.564-0.699) | 0.003 | 0.645(0.598-0.692) | <0.001 | 0.633(0.567-0.700) | <0.001 |
| Model3 | 0.627(0.588-0.666) | 0.001 | 0.586(0.526-0.646) | <0.001 | 0.632(0.593-0.671) | <0.001 | 0.585(0.526-0.644) | <0.001 |
| External validation cohort 1 | |  |  |  |  |  |  |  |
| Model1 | 0.706(0.653-0.759) |  | 0.752(0.681-0.822) |  | 0.705(0.652-0.758) |  | 0.752(0.682-0.822) |  |
| Model2 | 0.662(0.607-0.717) | 0.049 | 0.660(0.581-0.738) | 0.002 | 0.661(0.606-0.716) | 0.046 | 0.659(0.581-0.738) | 0.002 |
| Model3 | 0.604(0.555-0.653) | <0.001 | 0.580(0.510-0.651) | 0.001 | 0.603(0.552-0.654) | <0.001 | 0.580(0.510-0.651) | <0.001 |
| External validation cohort 2 | |  |  |  |  |  |  |  |
| Model1 | 0.743(0.690-0.796) |  | 0.763(0.689-0.837) |  | 0.742(0.685-0.799) |  | 0.754(0.677-0.830) |  |
| Model2 | 0.706(0.649-0.763) | 0.027 | 0.692(0.608-0.775) | 0.009 | 0.699(0.636-0.762) | 0.038 | 0.656(0.570-0.741) | 0.003 |
| Model3 | 0.649(0.594-0.704) | <0.001 | 0.626(0.546-0.706) | <0.001 | 0.642(0.585-0.699) | <0.001 | 0.605(0.527-0.684) | 0.059 |

Note: ^a^, DeLong.

Model1: Nomogram (Age, Differentiation status, Lauren type, Circulating exosomal lncRNA-GC1 and the AJCC stage);

Model2: Age, Differentiation status, Lauren type and the AJCC stage;

Model3: AJCC stage system.
