# Supplementary material for: Circulating exosomal gastric cancer-associated long noncoding RNA1 as a noninvasive biomarker for predicting chemotherapy response and prognosis of advanced gastric cancer: A multi-cohort, multi-phase study
Source: eBioMedicine. 2022 Mar 27;78:103971. doi: 10.1016/j.ebiom.2022.103971 (PMC8965144; doi:10.1016/j.ebiom.2022.103971)
Supplement: Supplementary file 10 [file mmc10.docx]

**eTable.9. Treatment interaction with circulating exosomal lncRNA-GC1 for disease-free and overall survival.**

| **Circulating exosomal lncRNA-GC1** | **Chemotherapy** | **No chemotherapy** | **HR (95% CI)** | **P value for interaction** |
| --- | --- | --- | --- | --- |
| **AJCC stage II GC** | | | | |
| **Disease-free survival** | | | | |
| High-level group (n=142) | 67 | 75 | 0.790 (0.515-1.211) | 0.005 |
| Low-level group (n=86) | 47 | 39 | 0.213 (0.088-0.512) |  |
| **Overall survival** |  |  |  |  |
| High-level group (n=142) | 67 | 75 | 0.796 (0.515-1.229) | 0.005 |
| Low-level group (n=86) | 47 | 39 | 0.135 (0.038-0.478) |  |
| **AJCC stage III GC** | | | | |
| **Disease-free survival** |  |  |  |  |
| High-level group (n=411) | 231 | 180 | 0.800 (0.632-1.012) | 0.029 |
| Low-level group (n=175) | 87 | 88 | 0.363 (0.226-0.585) |  |
| **Overall survival** |  |  |  |  |
| High-level group (n=411) | 231 | 180 | 0.805(0.634-1.022) | 0.018 |
| Low-level group (n=175) | 87 | 88 | 0.311 (0.174-0.557) |  |
